# Translation and Validation of the Greek Version of the Questionnaire for Assessing Fear of Radiotherapy in Oncology Patients

**DOI:** 10.7759/cureus.62801

**Published:** 2024-06-20

**Authors:** Apostolina Ouzouni, Georgios A Plataniotis, Antonio Capizzello, Areti Gkantaifi, Areti Tsaloglidou, Dimitra-Anna Owens, Maria Lavdaniti

**Affiliations:** 1 Clinical Pathology, American Hellenic Educational and Progressive Association, Thessaloniki, GRC; 2 Radiation Oncology, Aristotle University of Thessaloniki, Thessaloniki, GRC; 3 Nursing, International Hellenic University of Thessaloniki, Thessaloniki, GRC; 4 Psychiatry, Athens University Medical School, Athens, GRC

**Keywords:** oncology patients, radiotherapy, fear, questionnaire, validation, translation

## Abstract

This study aims to validate the Greek translation of the Questionnaire for Assessing Fear of Radiotherapy in Oncology Patients (QAFRT). Conducted as a cross-sectional pilot study, it involved 149 cancer patients from two radiotherapy departments in Thessaloniki, Greece. The sample included patients with various cancer types and stages, all of whom were undergoing radiation treatment. The QAFRT, originally containing 15 items measured on a Likert scale, was translated into Greek using the back-translation method. Exploratory factor analysis was performed on the translated version, resulting in a refined 13-item questionnaire encompassing four factors: fear of radiotherapy effectiveness, fear of illness during radiotherapy, fear of radiotherapy's impact on daily life, and fear of side effects and relationships. The reliability of the QAFRT was confirmed with Cronbach’s α of 0.82 and intraclass correlation coefficient coefficients ranging from 0.92 to 0.98. The study concludes that the Greek version of the QAFRT is a reliable and valid tool for assessing the fear of radiotherapy in cancer patients, highlighting the need for adequate psychological support for those with high levels of fear.

## Introduction

Cancer is one of the leading causes of death worldwide. According to the Global Cancer Observatory, the number of new cancer cases in 2022 was 19,976,499 worldwide. The number of cancer deaths in the same year was 9,743,832 worldwide. In Greece, there were 65,703 newly diagnosed cancer patients in 2022, and the number of deaths was 32,385 [1]. According to the Hellenic Statistical Authority, 26% of deaths in Greece in 2019 were attributed to cancer, making it the second-leading cause of death [2].

In over 50% of all cancer patients, radiotherapy is one of the main treatment modalities being utilized, either alone or more commonly in combination with surgery, chemotherapy, and hormone therapy, to treat a wide range of cancers [3]. Cancer patients have to cope with the stress of diagnosis and the conflicting and uncontrollable information they get from family, friends, and the media. Therefore, they usually develop reservations and fears regarding radiotherapy [4].

Cancer patients undergoing radiotherapy experience more anxiety compared to patients with other diseases. Psychological effects such as anxiety, anger, fatigue, depression, fear, physical and mental stress, eating disorders, pain, sexual problems, impaired social functioning, and financial difficulties are some of the experiences throughout the cancer pathway that can markedly impact the quality of life [5,6].

Fear is described as an unpleasant, often strong emotion caused by anticipation or awareness of danger and accompanied by increased autonomic activity. Moreover, fear has consistently been identified in the literature as a common problem with negative psychological and functional consequences [7]. Radiotherapy can cause psychological problems such as anxiety, depression, and social isolation caused by the fear of radiotherapy itself or its side effects [8]. Radiotherapy is associated with misunderstandings and fears. The mass media may contribute to patients' fears of radiation therapy by familiarizing the public with nuclear power and nuclear weapons while leaving them relatively uninformed about the medical uses of radiation [9].

According to Arikan et al., fear of radiotherapy machines, fear that something bad will happen during radiotherapy, and fear that radiotherapy will not work may all cause anxiety and depression in patients with cancer who undergo radiation therapy. Additionally, this study found that 27.1% of patients indicated that their anxiety stemmed from the fear of not being able to manage the side effects of radiotherapy [10].

The results of the study by Shaverdian et al. showed that almost half of the patients stated that they had previously read or heard frightening stories about the serious side effects of radiation. The most commonly cited initial fears of radiotherapy included damage to internal organs, skin burns, being radioactive, and being unable to perform everyday tasks. Other fears included fatigue and weakness, damage to the immune system, pain, changes in appearance, and cost. Only about 6% of patients stated that they were not afraid of radiotherapy. Additionally, this study illustrated how widespread the fears and misconceptions about breast radiotherapy are and showed that patients’ actual treatment experiences are usually better than their original expectations. Interestingly, the vast majority of patients agreed that their initial negative impressions and fears about breast radiotherapy were unfounded [11].

We understand that only a few studies related to the fear of radiation in cancer patients have been conducted. One of them is by Zivkovic Radojevic et al., who performed a prospective qualitative study based on the development, validation, and reliability testing of a questionnaire developed for assessing radiotherapy-induced fear in cancer patients. The results of the study showed that the questionnaire is a reliable and valid instrument for assessing the level of fear of radiotherapy in cancer patients [12].

To the best of our knowledge, in Greece, there have been no similar studies. This study aimed to validate the Greek translation of the Questionnaire for Assessing Fear of Radiotherapy in Oncology Patients (QAFRT) [12].

## Materials and methods

Study design

This study is a cross-sectional pilot study conducted in two radiotherapy departments: the first in a Greek university hospital and the second in an anticancer hospital in Thessaloniki.

Sample

The study included cancer patients with histopathologically confirmed cancer of any site. The sample consisted of 149 out of 154 patients; five cancer patients refused to answer the questionnaire and were diagnosed with cancer who attended the cancer outpatients or inpatients from April 2022 to September 2022. There were a variety of cancer diagnoses, comprising 21 different types of cancer. The criteria for inclusion in the study were as follows: patients aged 18 years or older, patients with verbal communication ability in spoken and written Greek language, any stage or localization of histologically verified carcinoma, receiving radiation treatment, and having no history of psychotic illness and/or dementia.

A total of 149 cancer patients participated according to the guidelines for the respondent-to-item ratio, which ranged from 5:1 (i.e., 50 respondents for a 10-item questionnaire), 10:1, to 15:1 or 30:1 [13]. There is a variation in the types of questionnaires that can be used, and there are no absolute rules for the appropriate sample size to validate a questionnaire. The Kaiser-Meyer-Olkin (ΚΜΟ) test showed that the sample size was satisfactory, as the KMO index (KMO=0.70) was above the 0.5 recommended by Kaiser and Rice [14].

Collection of data and measures

Demographic data, diagnoses, and clinical characteristics were obtained from patients’ medical records. The questionnaire used to assess the level of fear of radiotherapy in cancer patients was employed in this study [12]. We obtained written permission to use the questionnaire [12], which was translated into Greek using the back-translation method [15]. The final version of the questionnaire integrated 15 questions intended to measure fear of radiation therapy in general. Each question had five possible answers according to the Likert scale (quantitative rating scale), coded from 0 to 4; therefore, the total score was expected to be within the range of 0 to 60.

After the factor analysis, the questionnaire consisted of two factors. The first factor, "fear of patients related to the attitude of family and friends and the continuation of life after irradiation," included 10 questions. These questions pertain to the patient’s fear during and/or after radiotherapy, such as changing family, partners,’ and friends’ relationships, as well as fear related to daily activities and life after completing treatment.

The second factor, "fear linked to disease prognosis and the adverse effects of radiation," encompassed five questions. These questions were related to patients’ fear of lacking necessary information about the potential adverse effects of radiotherapy, the fear of disease progression and damage to other organs, and the fear of the radiation procedure itself.

The application of Zivkovic Radojevic et al.'s [12] questionnaire makes it possible to identify patients with elevated levels of fear of radiotherapy, and it is also unique. Patients with a high total score would require special attention and adequate psychological or psychiatric support, with a maximum reduction of adverse events, in order to allow the treatment to be implemented fully and adequately. Furthermore, the researcher informed the participants about the purpose of the study and remained by their side to answer any queries or give any indications during the questionnaire completion.

Ethical considerations

Written permission from the creators of the questionnaire was obtained from the research team who conducted the Greek translations for the use of the above-mentioned questionnaire [12]. Ethical approval in order to conduct the study was obtained from the scientific committee of the participating hospitals (approval number: 10035/23.02.2022) and the Ethics Committee of the International Hellenic University of Thessaloniki (approval number: 14/01.11.2022).

After the necessary information was given prior to the completion of the questionnaire, patients signed an informed consent form. Confidentiality of the collected data was ensured in accordance with Regulation (EU) 2016/679 of the European Parliament on the protection of natural persons with regard to the processing of personal data and on the free movement of such data, repealing Directive 95/46/EC (General Data Protection Regulation).

Statistics

To investigate the reliability of the scale (pilot and main study), Cronbach’s α internal consistency index and the intraclass correlation coefficient (ICC) were calculated for the reliability of repeated measurements. To investigate the factor structure of the scale, an exploratory factor analysis (EFA) was carried out on the Greek version of the scale. Principal axis factoring was applied as the factor extraction method, with varimax rotation used for better interpretation of the factors.

Before performing factor analysis, the ΚΜΟ indices for the adequacy of the sample size for the analysis and Bartlett’s test of sphericity were examined. The demographic and clinical characteristics of participants were described by absolute (N) and relative frequency values (%) for qualitative variables and means and standard deviations for quantitative variables.

Inductive statistics were employed to examine the effect of patient demographic and clinical characteristics on radiotherapy fear questionnaire scores. For comparisons between two groups, the t-test for independent samples was used, while for comparisons of more than two groups, the one-way ANOVA test was utilized. For statistically significant effects, multiple comparisons with significance criterion correction were applied employing the Sidak method. The significance criterion was defined as p<0.05 (two-tailed test). SPSS Statistics version 25 (IBM Corp. Released 2017. IBM SPSS Statistics for Windows, Version 25.0. Armonk, NY: IBM Corp.) and AMOS version 22 (IBM Corp., Armonk, NY) were used for the analysis.

## Results

Descriptive statistics were utilized to assess demographic and clinical characteristics. Qualitative variables are described as N (%), whereas continuous variables are presented as M±SD. The structure of the Greek version of the scale was assessed by conducting the EFA. Principal axis factoring with varimax rotation was used for EFA.

Cronbach’s α was calculated to assess the internal consistency of the scale, whereas the ICC was used to measure the temporal stability of the scale in a subset of participants. The normality of the distribution was assessed via the Lilliefors test. Quantitative demographic variables were transformed into qualitative variables (age, cancer duration, and number of sessions completed) by using percentiles as cut-offs (four groups). The effects of demographic and clinical characteristics on the fear of radiotherapy scale were assessed via independent t-tests (for two groups) or one-way ANOVA (for more than two groups). Significant effects were followed up by multiple comparisons with Sidak correction.

In total, 149 patients were included in the study, of whom 64.5% were males. The mean age was 66.20 ± 11.90 years old. Most participants were married (77.2%), and many were retired (63.1%). Various cancer types were observed in the sample, of which lung cancer accounted for 20.1% and breast cancer for 21.5%. The most common cancer stage was the second stage (40.3%). Moreover, a high percentage of patients underwent surgery as therapy (66.4%), whereas only 2.7% underwent biological therapy. Most participants reported going to radiotherapy with friends or family (79.9%). The mean number of radiotherapy sessions completed was 8.58 ± 8.50. The clinical and demographic characteristics of the samples are presented in Table 1.

**Table 1:** Clinical and demographic characteristics

Characteristics		n	%
Gender	Male	96	64.4
	Female	53	35.6
Age	Mean age (standard deviation) range	66.20 ± 11.90 (40-88)	
	40-56 years old	35	23.5
	57-65 years old	28	18.8
	66-75 years old	47	31.5
	76-88 years old	39	26.2
Family status	Married	115	77.2
	Non married	32	21.5
	Divorced	1	.7
	Widowed	1	.7
Education	Did not go to school	5	3.4
	Primary school	51	34.2
	Middle school	50	33.6
	High school	28	18.8
	Technical university	7	4.7
	Undergraduate degree	8	5.4
Employment status	Private sector employee	26	17.4
	Civil servant	14	9.4
	Freelancer	8	5.4
	Unemployed	7	4.7
	Retired	94	63.1
Type of cancer	Ca left flank	1	0.7
	Ca penile	1	0.7
	Ca tongue	1	0.7
	Ca glottic	1	0.7
	Ca skin	7	4.7
	Ca endometrial	4	2.7
	Ca mandibular	1	0.7
	Ca laryngeal	26	17.4
	Ca breast	32	21.5
	Ca kidney	1	0.7
	Ca esophageal	1	0.7
	Ca rectum	11	7.4
	Ca parotid gland	1	0.7
	Ca lung	30	20.1
	Ca prostate	22	14.8
	Ca stomach	2	1.3
	Ca lip	1	0.7
	Ca cervical	1	0.7
	Ca soft palate	3	2.0
	Ca pharynx	1	0.7
	Ca ear	1	0.7
Staging System	Clinical staging	56	37.6
	Pathological staging	93	62.4
Cancer stage	1^st^ stage	43	28.9
	2^nd^ stage	60	40.3
	3^rd^ stage	35	23.5
	4^th^ stage	11	7.4
Combination therapy	Yes	102	68.5
	No	47	31.5
Therapy type (multiple answers)	Surgery	73	66.4%
	Chemotherapy	49	44.5%
	Hormonal therapy	22	20.0%
	Biological therapy	3	2.7%
Comorbidity with other diseases	Yes	92	61.7
	No	57	38.3
Went to radiotherapy sessions with:	With others (friends, family, etc.)	119	79.9
	Alone	30	20.1
Duration of cancer since diagnosis (months)	Mean duration ± standard deviation (range)	4.17±2.84 (0.10-13.74) months	
	0-2.07 months	39	26.2
	2.13-3.64 months	36	24.2
	3.67-5.93 months	38	25.5
	5.97-13.74 months	36	24.2
Total duration of radiotherapy	Mean number of sessions ± standard deviation (range)	25.93±4.91 (10- 35)	
	≤25	77	51.7
	>25	72	48.3
Radiotherapy sessions completed	Mean number of sessions ± standard deviation (range)	8.58±8.50 (1-30)	
	1 session	26	17.4
	2 sessions	31	20.8
	3-15 sessions	52	34.9
	≥16 sessions	40	26.8

Before conducting the EFA, a correlation matrix was created to assess the connections between the items. Items 13 and 14 had a limited number of significant correlations with the other items and were removed from the analysis. All remaining items had appropriate correlations. No missing values were noticed in the questionnaire. Table 2 displays the descriptive characteristics of each item. Mean scores ranged between 2.31 and 3.05, whereas correlations were adequate.

**Table 2:** Mean, standard deviation, skewness, kurtosis, and Pearson correlation of the questions

Questions		Mean	Standard deviation	Skewness	Kurtosis	Pearson correlation
1	Do you have a fear that radiation can affect the appearance of a new tumor?	3.01	0.83	-0.53	-0.27	0.71
2	Are you afraid that radiation therapy can damage other organs, which are not subjected to radiotherapy?	3.05	0.73	-0.50	0.17	0.69
3	Do you have a fear that you will endanger your family because you are in radiation therapy?	2.64	0.73	0.23	-0.49	0.39
4	Are you afraid that radiotherapy will cause burns at the site of application of radiation?	2.87	0.69	-0.31	0.16	0.45
5	Are you afraid that radiation therapy will hinder your everyday activities?	2.38	0.60	0.59	0.15	0.37
6	Do you have a fear that friends will change their relationship with you because you are being treated with radiotherapy?	2.38	0.90	0.07	-0.78	0.53
7	Were you afraid when you were told that you would continue the treatment of radiation therapy?	2.95	0.82	-0.50	-0.17	0.25
8	Do you feel disturbed while expecting the application of radiotherapy?	2.57	0.95	-0.11	-0.87	0.46
9	Are you afraid that radiation therapy can cause permanent damage to the region of irradiation?	2.86	0.75	-0.24	-0.26	0.58
10	Do you have a fear that your partner will change their relationship with you because you are being treated with radiotherapy?	2.32	0.91	0.54	-0.45	0.21
11	Do you think more often than usual about your illness while on radiation therapy?	3.01	0.88	-0.49	-0.59	0.32
12	Do you have a fear that radiation therapy will not be effective against your illness?	3.03	0.80	-0.52	-0.18	0.59
15	Are you preoccupied with thinking about radiotherapy during the whole day?	3.11	0.97	-0.99	0.09	0.54

**Figure 1:**
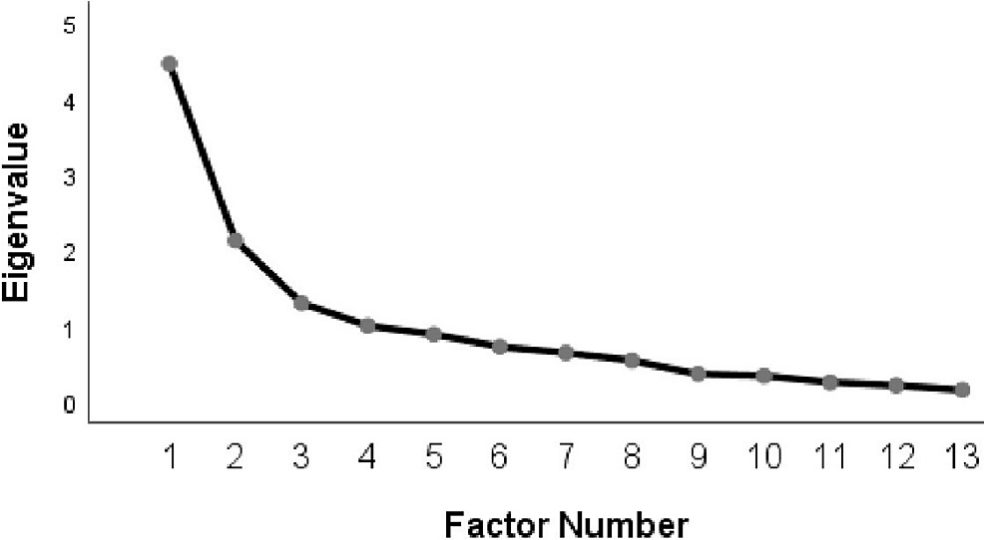
Scree plot of the data

A principal axis factor analysis was conducted on the 13 remaining items with varimax rotation. The ΚΜΟ measure (KMO=0.70) was higher than the suggested cut-off of 0.5 [14], indicating an adequate sample size. Bartlett’s test of sphericity yielded a statistically significant result (χ2 (78)=839.37, p<0.001), indicating that the data were suitable for the factor analysis. According to Kaiser’s criterion for retaining factors, four factors had eigenvalues over Kaiser’s criterion of 1 and collectively explained 68.35% of the total variance. The generated scree plot (Figure 1) shows a discrete inflection point after the fourth factor.

Table 3 displays the factor loadings after rotation, presenting only loadings over 0.3. The first factor comprises items 6, 8, 9, and 12; the second factor comprises items 1 and 11; the third factor comprises items 4, 7, and 15; and the fourth factor comprises items 2, 3, 5, and 10.

**Table 3:** Rotated component matrix (principal component analysis with varimax rotation)

Questions		Factors			
		1	2	3	4
1	Do you have a fear that radiation can affect the appearance of a new tumor?	0.498	0.661		
2	Are you afraid that radiation therapy can damage other organs, which are not subjected to radiotherapy?		0.442	0.350	0.586
3	Do you have a fear that you will endanger your family because you are in radiation therapy?				0.387
4	Are you afraid that radiotherapy will cause burns at the site of application of radiation?			0.535	
5	Are you afraid that radiation therapy will hinder your everyday activities?				0.744
6	Do you have a fear that friends will change their relationship with you because you are being treated with radiotherapy?	0.658			0.330
7	Were you afraid when you were told that you would continue the treatment of radiation therapy?			0.656	
8	Do you feel disturbed while expecting the application of radiotherapy?	0.619			
9	Are you afraid that radiation therapy can cause permanent damage to the region of irradiation?	0.570			
10	Do you have a fear that your partner will change their relationship with you because you are being treated with radiotherapy?	0.363			0.403
11	Do you think more often than usual about your illness while on radiation therapy?		0.824		
12	Do you have a fear that radiation therapy will not be effective against your illness?	0.787	0.306		
15	Are you preoccupied with thinking about radiotherapy during the whole day?		0.412	0.645	

In terms of reliability, Table 4 displays Cronbach’s α coefficient for the questionnaire factors and the total scale score. The scale demonstrated satisfactory reliability, with a Cronbach’s α of 0.82 [16]. The α coefficients for the factors ranged from 0.62 to 0.78. To evaluate the questionnaire’s temporal stability, a randomly selected subset of 43 participants underwent retesting at least a month after the initial measure. The ICC coefficients, ranging between 0.92 and 0.98, indicated excellent reliability (Table 5) [17].

**Table 4:** Internal consistency of the scale

Factor	Cronbach’s α
1	0.78
2	0.72
3	0.68
4	0.62
Total scale	0.82

**Table 5:** Temporal stability of the questionnaire

Factor	ICC	95% CI	p-value
1	0.98	0.96-0.99	<0.001
2	0.92	0.86-0.96	<0.001
3	0.94	0.89-0.96	<0.001
4	0.94	0.89-0.97	<0.001
Total scale	0.93	0.88-0.96	<0.001

Table 6 displays the descriptive characteristics of both the questionnaire’s total score and its subscales. The mean total score was 36.16 ± 6.01, while the mean scores for the subscales ranged from 6.02 to 10.83.

**Table 6:** Descriptive of the fear of radiotherapy scale scores

Factor	Minimum	Maximum	Mean	Standard deviation
Total scale	19.00	46.00	36.16	6.01
1	4.00	16.00	10.83	2.66
2	2.00	8.00	6.02	1.51
3	4.00	12.00	8.92	1.95
4	6.00	15.00	10.39	2.06

The effects of demographic and clinical characteristics on the fear of radiotherapy were assessed. There were no significant effects of age categories, family status, cancer stage, and type of therapy (combination or not) (all ps=ns). Table 7 shows all the significant effects of the demographic characteristics. The analysis demonstrated that males had a higher score in factor 4 than females (p=0.03). Participants who were supported during radiotherapy scored higher in factor 4 than those who completed the sessions with minimal support and assistance (p=0.01). The time elapsed since the cancer diagnosis had a significant effect (p=0.02). Follow-up analysis with Sidak correction revealed that patients with 0-2.07 months of cancer duration had a higher score in factor 4 than those with 2.13-3.64 months and those with 3.67-5.93 months of cancer duration. There were also significant effects of the radiotherapy duration in factors 1 (p=0.04) and 2 (p=0.03). However, the multiple comparisons did not reveal statistically significant differences across the four groups (1 session, 2 sessions, 3-15 sessions, ≥16 sessions).

**Table 7:** Significant effects of demographic characteristics on the fear of radiotherapy scale scores t-test for independent sample, one-way ANOVA * Welsh's F test Superscripted letters correspond to significant results after multiple comparisons with Sidak corrections: ^a ^Statistically significant difference with the group "0-2.07 months," ^b ^Statistically significant difference with the group "2.13-3.64 months," and ^c ^Statistically significant difference with the group "3.67-5.93 months"

Scale	Demographic	Mean	Standard deviation	t (df) or F(df)	p-value
Factor 4					
Gender	Male	10.6	1.91	2.16 (147)	0.03
	Female	9.91	2.23		
On radiotherapy sessions	Alone	9.40	2.14	-3.03 (147)	0.01
	With others	10.64	1.96		
Duration of cancer diagnosis	a. 0-2.07 months^b, c^	11.26	1.63	3.61 (1.148)^*^	0.02
	b. 2.13-3.64 months^a^	9.89	2.00		
	c. 3.67-5.93 months^a^	10.03	1.75		
	d. 5.97-13.74 months	10.33	2.55		
Factor 1					
Number of sessions completed	1 session	11.19	2.37	2.94 (3.148)^*^	0.04
	2 sessions	11.81	2.10		
	3-15 sessions	10.56	2.73		
	≥16 sessions	10.20	2.98		
Factor 2					
Number of sessions completed	1 session	6.46	1.30	3.16 (3.148)^*^	0.03
	2 sessions	6.48	1.23		
	3-15 sessions	5.71	1.66		
	≥16 sessions	5.78	1.53		

## Discussion

To the best of our knowledge, the present study is the first published scientific work in Greece on the weighting of a questionnaire in Greek to assess the feelings of fear among cancer patients undergoing radiation therapy. The reliability of the specific tool is considered satisfactory, with Cronbach’s α value for the measurement scale being α=0.82, while the α coefficient ranged from 0.62 to 0.78. Furthermore, the ICC coefficients ranged from 0.92 to 0.98. Thus, the results of the study confirm that the QAFRT in cancer patients is a reliable and valid instrument for measuring the level of fear of radiotherapy.

The questionnaire consists of 15 questions, and to investigate the validity of the conceptual construct, an EFA was performed. Prior to applying the EFA, correlations between the questions were calculated, as this is one of the assumptions. Generally, the questions exhibited satisfactory correlations with each other. However, questions 13 and 14 showed few statistically significant correlations with the other questions, with almost all correlation coefficients being below 0.3. Consequently, items with low correlations with the total questionnaire score should be either discarded or revised [13]. Furthermore, in the original questionnaire used to create the QAFRT, it was noted that two questions correlated poorly with other items and were thus eliminated [12]. The criteria for elimination were questions with correlation coefficient values below 0.2. Consequently, these two questions were removed from the analysis, leaving 13 questions for the EFA. No missing values were observed.

A principal axis factor analysis was conducted on the 13 remaining items with varimax rotation. The KMO measure (KMO=0.70) was higher than the suggested cut-off (0.5) [13], indicating an adequate sample size. Bartlett’s test of sphericity showed a statistically significant result (χ2 (78)=839.37, p<0.001), revealing that the data were eligible for the factor analysis. According to Kaiser’s criterion for retaining factors, four factors had eigenvalues over Kaiser’s criterion of 1, which in combination explained 68.35% of the total variance.

The first identified factor, termed "the fear of the effectiveness of radiotherapy," consisted of four questions and demonstrated high internal consistency with α=0.78. The second factor, "the fear of illness during radiotherapy," comprised two questions and exhibited α=0.72. The third factor, labeled "the fear of the effect of radiotherapy on daily life," comprised three questions with a Cronbach’s α of 0.68. The fourth factor, "the fear of side effects and relationships with family and friends," had α=0.62. The original version featured two factors: the first, titled "fear of patients related to the attitude of family and friends and the continuation of life after irradiation," and the second, named "fear linked to disease prognosis and the adverse effects of radiation."

Loneliness is a known risk factor for poor mental health in the general population [18]. In our study, participants who were supported during radiotherapy had higher scores in factor 4 than those who did the sessions alone (p=0.01). Gimson et al. found that emotional support from family and/or friends, both in the hospital and at home, was an important way of lessening anxiety. Some participants reported experiencing less anxiety when family and/or friends helped them with hospital appointments [5]. On the other hand, according to a supporting study, cancer patients harbored fears of relying on caregivers [19]. The fear of dependency was one of the reasons leading to suicidal thoughts and exacerbating the patient’s psychological well-being.

In our study, we observed a significant main effect of cancer duration (p=0.02). Follow-up analysis employing Sidak correction indicated that patients with a cancer duration of 0-2.07 months exhibited higher scores in factor 4 compared to those with durations of 2.13-3.64 months and 3.67-5.93 months. Additionally, significant effects of radiotherapy duration were found on factors 1 (p=0.04) and 2 (p=0.03).

Recently, Piroth et al. [20] utilized the State-Trait Anxiety Inventory in combination with the Beck Depression Inventory - Fast Screen [21], which assesses depression symptoms, the Hamilton Anxiety and Depression Scale [22] for a combined assessment of anxiety and depression symptoms, and the Visual Analogue Emotional Scale [23] for the assessment of rapidly changing emotional states to investigate feelings of fear and anxiety in female breast cancer patients undergoing radiation therapy.

The primary findings revealed that the highest levels of fear and anxiety related to radiation therapy occurred during its initial stages, whereas the lowest levels were observed toward the end of treatment. Additionally, both anxiety and depression were more pronounced in women with a history of multiple comorbidities and chronic diseases. In our study, significant effects of radiotherapy duration were noted in factors 1 (p=0.04) and 2 (p=0.03). However, multiple comparisons did not reveal statistically significant differences across the four groups (1 session, 2 sessions, 3-15 sessions, ≥16 sessions).

Gimson et al. [5] found that cancer patients undergoing radiotherapy felt fear regarding whether certain side effects might be permanent. In addition, this study has shown that previous experience with the cancer pathway can sometimes increase fear of the unknown.

As research continues to globalize, scholars increasingly need to validate and translate established scales into languages other than those in which the scales were originally developed. Back-translation is the dominant procedure for translating scales and has been widely used in medical research [24]. This survey focuses on the translation, back-translation, and validation of a questionnaire assessing the fear of radiation in cancer patients.

Limitations

Although the current study shares the experiences of 149 cancer patients treated with radiotherapy, it has several limitations. Despite its diversity, the population in the current study was limited to patients from two hospitals and may therefore be biased by geographic and socioeconomic influences. Another limitation is that patients with serious conditions, such as those with an altered level of consciousness, were not evaluated. However, it boasts several strengths, notably being the first survey in Greece to deal with the translation and validation of a questionnaire regarding the fear of radiotherapy in cancer patients. Regarding clinical applicability, this questionnaire has positive aspects for assessing the fear of radiotherapy in cancer patients, such as the small number of items and the short time required to answer the questionnaire. Thus, Greek professionals working in oncology units can utilize this tool to recognize, provide for, monitor, and even prevent fear symptoms, thereby enhancing the healthcare provided.

## Conclusions

The questionnaire serves as a valid and reliable tool for evaluating the fear of radiation among Greek-speaking cancer patients. Patients exhibiting a high total score of fear necessitate specialized support. It’s imperative to establish personalized supportive care at the onset of radiation therapy, addressing their specific sensitivities and anxiety reactions. This tailored approach aims to mitigate additional psychological and physical stress effectively. Therefore, it is crucial for healthcare professionals to closely monitor and assess the mental well-being of these patients by adopting a patient-centered approach. Healthcare providers can help improve the overall quality of life of cancer patients and enhance their treatment outcomes.
